# Low affinity glucocorticoid binding site ligands as potential anti-fibrogenics

**DOI:** 10.1186/1476-5926-8-1

**Published:** 2009-05-11

**Authors:** Carylyn J Marek, Karen Wallace, Elaine Durward, Matthew Koruth, Val Leel, Lucy J Leiper, Matthew C Wright

**Affiliations:** 1 Institute of Medical Sciences, University of Aberdeen, Foresterhill, Aberdeen, UK; 2 Institute of Cellular Medicine, Newcastle University, Framlington Place, Newcastle Upon Tyne, UK

## Abstract

**Background:**

Pregnane X receptor (PXR) agonists inhibit liver fibrosis. However, the rodent PXR activator pregnenolone 16α carbonitrile (PCN) blocks, *in vitro*, hepatic stellate cell-to-myofibroblast trans-differentiation and proliferation in cells from mice with a disrupted PXR gene, suggesting there is an additional anti-fibrogenic drug target for PCN. The role of the low affinity glucocorticoid binding site (LAGS) – which may be identical or associated with the progesterone receptor membrane component 1 (PGRMC1) – in mediating this anti-fibrogenic effect has been examined, since binding of dexamethasone to the LAGS in liver microsomal membranes has previously been shown to be inhibited by PCN.

**Results:**

Quiescent rat and human hepatic stellate cells (HSC) were isolated from livers and cultured to generate liver myofibroblasts. HSC and myofibroblasts expressed PGRMC1 as determined by RT-PCR and Western blotting. Quiescent rat HSC also expressed the truncated HC5 variant of rPGRMC1. Rat PGRMC1 was cloned and expression in COS-7 cells gave rise to specific binding of radiolabelled dexamethasone in cell extracts that was inhibited by PCN, suggesting that PGRMC1 may be identical to LAGS or activates LAGS binding activity. Liver microsomes were used to screen a range of structurally related compounds for their ability to inhibit radiolabelled dexamethasone binding to rat LAGS. These compounds were also screened for their ability to activate rat and human PXR and to inhibit rat HSC-to-myofibroblast trans-differentiation/proliferation. A compound (4 androstene-3-one 17β-carboxylic acid methyl ester) was identified which bound rat LAGS with high affinity and inhibited both rat and human HSC trans-differentiation/proliferation to fibrogenic myofibroblasts without showing evidence of rat or human PXR agonism. However, despite potent anti-fibrogenic effects *in vitro*, this compound did not modulate liver fibrosis severity in a rat model of liver fibrosis. Immunohistochemical analysis showed that rat liver myofibroblasts *in vivo *did not express rPGRMC1.

**Conclusion:**

LAGS ligands inhibit HSC trans-differentiation and proliferation *in vitro *but show little efficacy in inhibiting liver fibrosis, *in vivo*. The reason(s) for this disparity is/are likely associated with an altered myofibroblast phenotype, *in vitro*, with expression of rPGMRC1 *in vitro *but not *in vivo*. These data emphasize the limitations of *in vitro*-derived myofibroblasts for predicting their activity *in vivo*, in studies of fibrogenesis. The data also demonstrate that the anti-fibrogenic effects of PCN *in vivo *are likely mediated entirely *via *the PXR.

## Background

Liver fibrosis is a common response to chronic liver damage that at present does not have a therapeutic option yet. The predicted increase in chronic liver disease (*e.g*., hepatitis C infection, non alcoholic steatohepatitis) means that liver fibrosis will be an increasing clinical problem in the future [1]. Liver fibrosis is primarily dependent on the proliferation and activity of myofibroblasts typically identified through their expression of α-smooth muscle actin [1]. These cells are derived from the trans-differentiation of hepatic stellate cells (HSC) in response to damage although they may also be generated from the trans-differentiation of other cell types [1]. Nonetheless, the liver myofibroblast is primarily responsible for the production of much of the extracellular matrix proteins that constitute the fibrotic scarring in fibrosis as well as the factors which promote further proliferation and scar accumulation [1]. The process of trans-differentiation and resolution (reversal) of fibrogenesis is dependent on other cells types, notably leucocytes – which are recruited to sites of injury – and resident macrophages (Kupffer cells) [2]. These cells produce a range of cytokines that modulate the behaviour of myofibroblasts and may ultimately regulate the process of fibrosis.

Nuclear receptors are transcription factors frequently controlled by the binding of ligands. The pregnane X receptor (PXR) is a nuclear receptor whose transcriptional function is regulated by pregnane steroids, bile acids and some drugs [3-5]. The rodent PXR ligand pregnenolone 16α carbonitrile (PCN) inhibits liver fibrogenesis in rodents [6,7] and similar effects are seen with human PXR activators and human myofibroblasts, *in vitro *[8]. The role of the PXR in the PCN-dependent inhibition of liver fibrosis was confirmed using mice with a disrupted PXR gene [6]. However, HSC trans-differentiation, *in vitro*, was still inhibited by PCN despite an absence of PXR expression within the cells (as determined by RT-PCR) and in HSCs isolated from mice with a disrupted gene [6]. Previous work has shown that PCN competes with the specific-saturable binding of progesterone or the synthetic glucocorticoid dexamethasone to "low affinity glucocorticoid binding site" (LAGS) protein in rat liver microsomes [9-11]. We therefore hypothesized that an additional target for PCN in liver myofibroblasts is the LAGS.

The identity of the LAGS has yet to be determined although it shows similar – but not identical binding characteristics – to a steroid binding activity to which the progesterone receptor membrane component 1 (PGRMC1) may be associated [10-14]. There are 2 PGRMC genes in humans and rodents that code for ~28 kDa proteins. The proteins have a single N-terminal membrane spanning domain and do not show significant homology with other gene super-families such as nuclear receptors [12].

PGRMC1 has been shown to bind haem [13] but it remains contentious as to whether the protein directly binds steroids, as suggested by Peluso *et al *[14], or is a component of a complex that binds steroids. Our data with the human PGRMC1 suggest that phosphorylation of the protein or a component of the binding complex may be important for efficient steroid binding and may explain the difficulties of reconstituting steroid binding, when the protein is purified or over-expressed in mammalian cells [12]. Nonetheless, these data are limited and the identity of the binding protein remains to be unambiguously demonstrated.

Recent evidence suggests, however, that PGRMC1 binds to cytochrome P450s and functions to facilitate cytochrome P450-mediated metabolism of sterol biosynthesis [15]. Interestingly, PGRMC1 stably binds to cytochrome P450 51A1 [15], an isoform that has been shown to be expressed in activated human liver myofibroblasts [16].

We therefore hypothesized that PCN mediates its PXR-independent mechanism of inhibiting myofibroblast trans-differentiation/proliferation *via *a LAGS/PGRMC interaction. To test this hypothesis, rat PGRMC1 was cloned and expressed and binding of PCN to the protein or a complex containing this protein confirmed. Through a series of established *in vitro *screens, a putative ligand for rat and human PGRMC1-associated complex – that was not also a PXR activator – was identified and shown to potently inhibit rat and human liver myofibroblast trans-differentiation and proliferation, *in vitro*. However, this compound failed to show any anti-fibrogenic activity in an *in vivo *model of liver fibrosis because the target PGRMC1 was not expressed by myofibroblasts, *in vivo*.

## Results

### The PGRMC1 is expressed in rat and human HSCs and myofibroblasts

**Figure 1:**
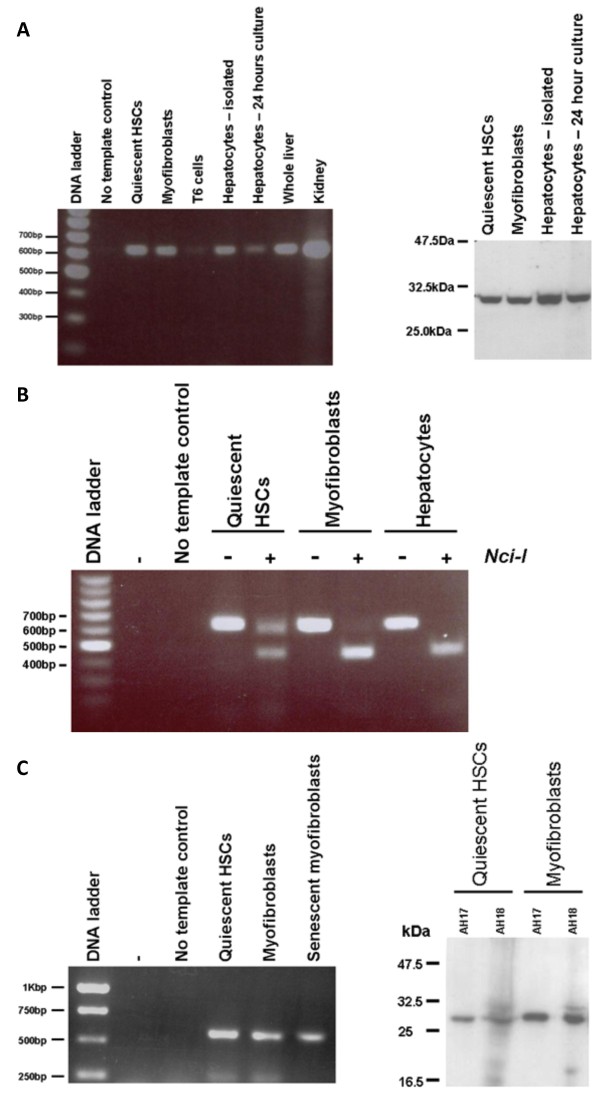
**Rat and human HSCs and myofibroblasts express PGRMC1 *in vitro***. Left panel, RT-PCR analysis for rPGRMC1 in rat cells and tissues as indicated using primer sequences and conditions as outlined in methods section. T6 cells are a rat hepatic stellate cell line [47] (a). Right panel, Western blot of the indicated cell types for rPGRMC1 using the anti-IZAb (a). RT-PCR products from the indicated cell types with and without digestion with the restriction enzyme *Nci-I *as indicated. rPGRMC1 PCR product does not contain an Nci-I site whereas the truncated HC5 variant contains a single site and is cleaved [17] (b). Left panel, RT-PCR analysis for hPGRMC1 in human cells using primer sequences and conditions as outlined in methods section. Senescent myofibroblasts had ceased proliferation (typically at passage 3–5) (c). Right panel, Western blot of the indicated anonymised donor cells for hPGRMC1 using the anti-IZAb (c). Results typical of a least 3 independent experiments and/or animals except right panel (c), 2 separate human donors.

Quiescent HSCs were isolated from normal rat liver or from histologically normal margins of human liver tissue resected because of the presence of a secondary tumour. When placed in the appropriate culture conditions, these cells trans-differentiate into myofibroblasts, reminiscent of the process that occurs in the liver in response to chronic liver damage [1]. Figure 1a shows that both quiescent rat HSCs and myofibroblasts expressed rPGRMC1 mRNA and protein at similar levels to rat hepatocytes. Quiescent rat HSCs also expressed the HC5 truncated variant of rPGRMC1 previously identified in kidney and blood [17] although expression was repressed and undetectable in myofibroblasts (Fig. 1b). Figure 1c shows that both quiescent human HSCs and myofibroblasts from 2 individuals expressed hPGRMC1 mRNA and protein, with an increased expression in myofibroblasts compared to the quiescent HSCs they were derived from.

### Expression of the rat progesterone receptor membrane component 1 (rPGRMC1) leads to steroid binding activity that interacts with PCN

It has been known for many years that the liver expresses LAGS activity [9-11,18-20]. Affinity purification of steroid binding proteins suggests that this activity is associated with the PGRMC1 protein (originally termed ratp28 [21], 25-Dx [22] or IZA [23] in rat and hpr6.6 in the human [24] on the basis of limited N-terminal amino acid sequencing).

**Figure 2:**
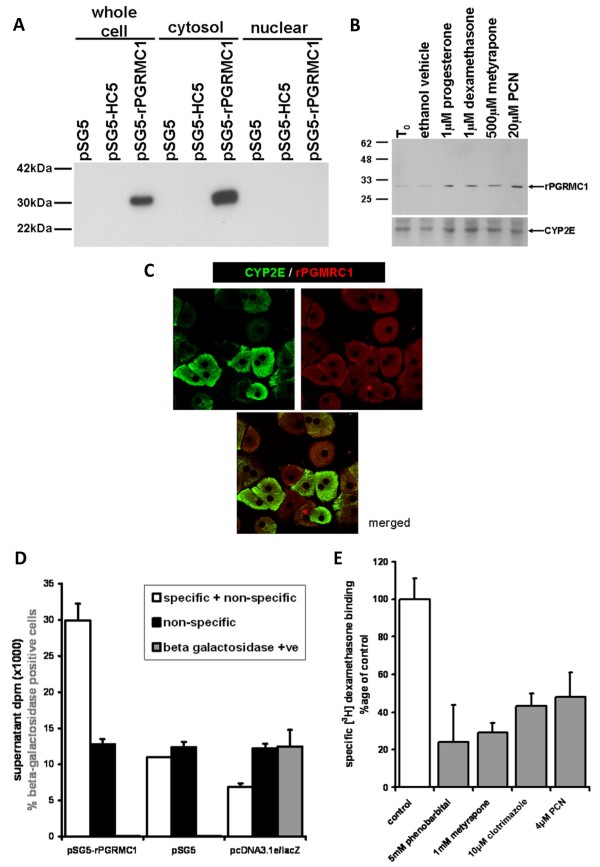
**Expression of rPGRMC1 results in dexamethasone binding activity**. Western blot for rPGRMC1 in various cell fractions using the anti-IZ Ab in COS-7 cells transfected with the indicated construct. All lanes were loaded with 10 μg protein/lane. Note, HC5 is a truncated form of rPGRMC1 cloned from rat kidney [17] (a). Western blot for rPGRMC1 using the anti-IZ Ab and CYP2E1 (lower blot). Rat hepatocytes were cultured for 24 hours to allow attachment (T_0_) and then treated for 24 hours with the indicated ligand or ethanol vehicle prior to analysis. Each lane contains 10 μg total protein/well, typical of 3 separate experiments (b). Confocal microscopy of rat hepatocytes demonstrating non-nuclear location of PGRMC1 and CYP2E1 (c). 200 × 10^6 ^COS-7 cells were transfected with pSG5-rPGRMC1, pSG5 or pcDNA3.1e/lacZ and 13,000 g cell extracts prepared and incubated with radiolabelled dexamethasone as outlined in methods section. Supernatant dpm after charcoal dextran treatment to remove free radioligand is given in dpm after normalisation of protein for total (specific and non-specific) – white bars; and non specific (by co-incubation of 1000-fold molar excess unlabelled dexamethasone) – black bars. The percentage of cells that stained positive for beta galactosidase activity (grey bars) was determined *in situ *in separate wells by examining at least 5 randomly selected low power fields. Data are the mean and standard deviation of at least 3 separate determinations from the same experiment, typical of 2 separate experiments (d). 200 × 10^6 ^COS-7 cells were transfected with pSG5-rPGRMC1. Dexamethasone binding activity was determined in whole COS-7 cells as outlined in methods section and in the presence of the indicated concentration of unlabelled potential competitor. Specific binding was determined by co-incubation of replicates also containing unlabelled 1000-fold molar excess of unlabelled dexamethasone. Typically, non specific binding accounted for between 40–60% of total binding of radioligand. Data are the mean and standard deviation of 3 separate determinations from the same experiment, typical of 3 separate experiments. Control is the mean and standard deviation specific activity of 3 determinations from the same experiment after subtraction of non-specific binding. The percent of binding in the presence of unlabelled competitors was determined after subtraction of non-specific binding. Data are typical of at least 2 separate experiments (e).

To formally test whether the expression of rPGRMC1 leads to the presence of a steroid binding activity, the full length cDNA for rPGRMC1 was cloned from rat myofibroblasts and expressed in COS-7 cells. Figure 2 demonstrates that the pSG5-rPGRMC1 construct directed the expression of a protein of approximately 28 kDa that accumulated in extra-nuclear cell fractions (Fig. 2a). The antibody employed also detected a protein of 28 kDa in hepatocytes which was up-regulated by several LAGS ligands (Fig. 2b) and was located in the extra-nuclear compartment (Fig. 2c). Receptor-ligand binding studies indicate that specific binding of dexamethasone was observed in COS-7 cells transfected with the pSG5-rPGRMC1 construct but not in cells transfected with an empty (pSG5) or pcDNA3.1e-LacZ vector (Fig. 2d). Therefore, the rPGRMC1 gene encodes a protein that either binds dexamethasone or combines with COS-7 proteins to form a dexamethasone binding complex.

Competition studies with cold potential competitors were performed to determine whether the rPGRMC1-associated binding activity also binds PCN. Although expression of rPGRMC1 was highly effective in COS-7 cells, the reliable detection of dexamethasone binding activity required such high amounts of transfected total COS-7 cell protein, that it was not feasible to perform wide ranging studies to determine affinities of dexamethasone and competitors. However, PCN as well as several other compounds, previously reported to compete with dexamethasone for binding to rat liver microsomes [9], were ligands on the basis of significant competition with dexamethasone for binding to the COS-7 cell extracts in which rPGRMC1 protein was over-expressed (Fig. 2e).

### Identifying novel ligands for the rPGRMC1-associated binding site activity through LAGS binding site activity

**Figure 3:**
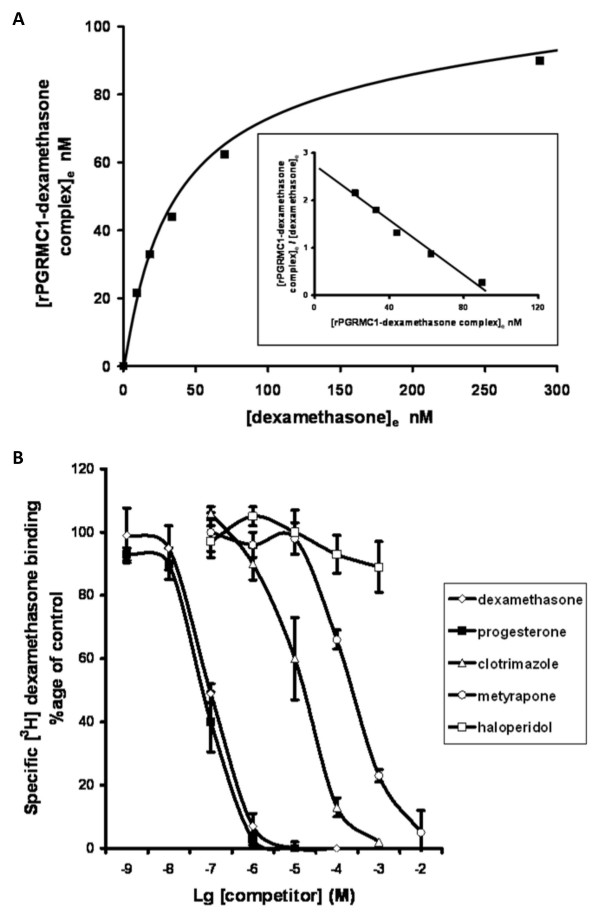
**Radiolabelled dexamethasone interacts in a specific and saturable manner with rat liver microsomes and binding is competed by selected compounds**. Male rat liver microsomes were incubated in duplicate with increasing concentrations of radiolabelled dexamethasone (ligand) with or without excess unlabelled dexamethasone and allowed to reach equilibrium on ice. A small volume of each incubation was removed to determine the total ligand concentration ([L_0_]) prior to removal of free unbound ligand by dextran/charcoal adsorption. Specifically bound ligand at equilibrium ([LR_e_]) was calculated by subtracting radioactive counts present in samples which also contained excess unlabelled dexamethasone after dextran-charcoal adsorption (and was typically < 5%). Free ligand concentration at equilibrium was calculated by subtracting specifically bound ligand at equilibrium from the total ligand concentration (i.e. [L_0_] - [LR_e_]) and assumes receptor-ligand stoichiometry of 1:1. Results typical of six separate preparations (a). Male rat liver microsomes were incubated with 50 nM [^3^H] dexamethasone as outlined in methods section with or without excess unlabelled dexamethasone (to determine non-specific binding) or a range of unlabelled compounds (added with ethanol vehicle such that final ethanol concentration was 1%, also present in controls). After overnight incubation on ice, free ligand was removed by dextran-charcoal adsorption and specifically bound radiolabelled dexamethasone determined (b).

The low expression of binding site activity in rPGRMC1-transfected COS-7 cells and relatively high level of non-specific binding in extracts (~50% of specific and non-specific binding), precluded this system from extensive and effective screening for novel rPGRMC1 ligands. However, the binding of dexamethasone to rat liver microsomes (LAGS activity) gave reproducible saturable binding characteristics with a k_D _of 51 nM and maximal binding site concentration of 8.3 pmoles/mg of microsomal protein (Fig. 3a); was subject to relatively low non-specific binding (~5% of specific and non-specific binding); was sufficiently abundant and binding was competed by progesterone and a range of other ligands (Fig. 3b, Table 1), but not by the sigma receptor ligand haloperidol [25]. Early work by Meyer *et al *identified a progesterone binding protein in pig liver microsomes with no competition for binding by dexamethasone (IC_50% _> 100 μM) [26], but competition by haloperidol [27]. There may be species differences between pig and rat which makes comparison complicated. However, a sigma-related binding site has been shown to be expressed in rat liver microsomes, which binds both progesterone and haloperidol [28]. Our data suggest that dexamethasone and progesterone share a binding site in rat liver microsomes, but on the basis that there is no competition for binding by haloperidol, this is not the sigma-related binding site. Therefore, the use of dexamethasone as a ligand for the LAGS is preferred over progesterone.

**Table 1:** IC_50% _values for competing radiolabelled dexamethasone from specific binding to rat liver microsomes.

**Cold Competitor**	**IC_50% _(10^-6 ^M)**
dexamethasone	0.098 ± 0.003
progesterone	0.081 ± 0.010
clotrimazole	40 ± 12
metyrapone	310 ± 52
haloperidol	> 10000

Data are the mean and standard deviation of at least 3 separate microsomal (isolated from different animals) determinations.

A range of substituted progestins were consequently screened for their ability to compete with dexamethasone for binding to rat liver microsomes and the results demonstrate binding of progestins was critically dependent on the presence of a keto group at position 3 (Additional file 1). Substituting the hydrogen at position 6 with bulkier groups markedly reduced affinity, whereas substitution of the hydrogen at position 11 had less effect on LAGS binding (Additional file 1). Alterations at position 17 also appeared to have less effect on affinity as long as the C17 chain was 1 or 2 carbons in length (Additional file 2).

The position of the methyl group in dexamethasone was critical for binding to LAGS, since betamethasone – which only differs from dexamethasone in the configuration of the methyl group at position 16 – had an approximately 100 fold lower affinity for binding (Additional file 2). The moieties at position 17 also appear to be important for dexamethasone binding, since both small and bulky group substitution prevented binding (Additional file 2).

### Screening rPGRMC1-associated binding site activity/LAGS ligands for PXR agonism in rat and human hepatocytes

**Figure 4:**
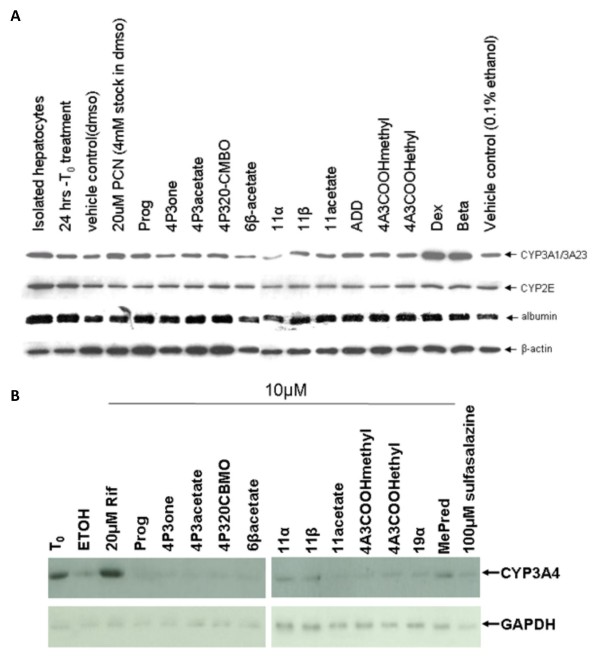
**Screening for PXR activators in rat and human hepatocytes via CYP3A induction**. Rat hepatocytes were isolated and cultured as outlined in methods section. After 24 hours of culture (T_0_), hepatocytes were treated for a further 24 hours with 10 μM of the indicated compound from a 1000 fold ethanol-solvated stock (except PCN, which was added to give 20 μM from a DMSO-solvated stock). Equivalent ethanol (0.1% v/v) and DMSO (0.5% v/v) vehicles are included. Cells were then analyzed for expression of the indicated protein by Western blotting, 10 μg total protein/lane. Results are typical of at least 3 separate experiments (a). Human hepatocytes were treated essentially as for rat hepatocytes except that all compounds were prepared as ethanol solvated stocks. Cells were then analyzed for expression of the indicated protein by Western blotting, 20 μg total protein/lane. Results are from one donor (LH2), typical of 2 different donors (b).

The canonical function of the PXR is a ligand-dependent transcriptional regulation of cytochrome P450 3A (CYP3A) genes, notably hepatic CYP3A1/3A23 and CYP3A4 genes in rat and human hepatocytes, respectively [4,5]. Screening the panel of ligands for CYP3A induction showed that the classic rat PXR activators PCN, dexamethasone and betamethasone induced CYP3A1/3A23 expression in rat hepatocytes (with no affect on CYP2E expression as expected [6]), whereas none of the other compounds markedly affected levels relative to untreated controls (Fig. 4a). In human hepatocytes, the potent human PXR activator rifampicin induced CYP3A4 expression as previously reported [29], whereas none of the other compounds showed any evidence of induction except methylprednisolone (Fig. 4b).

### Screening rPGRMC1 associated binding site activity/LAGS ligands for their ability in inhibit rat and human HSC trans-differentiation/proliferation into myofibroblasts

**Figure 5:**
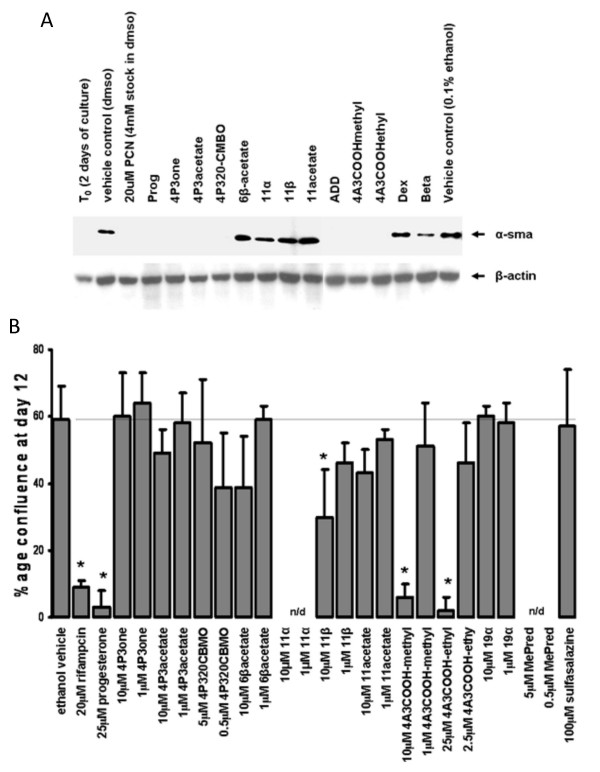
**Screening for inhibitors of trans-differentiation in rat and human HSCs – Part 1**. Rat HSCs were isolated and cultured for 2 days (T_0_) whereupon cells were treated with the indicated compound as outlined in methods section. After 9 days, cells were analyzed by Western blotting for α-smooth muscle actin (α-sma). Each lane contains 10 μg total protein/lane, results typical of at least 3 separate experiments (a). Human HSCs were treated with the indicated compound and confluence determined in randomly selected fields. Data are the mean and standard deviation confluence at day 12 of 3 separate treatment dishes from the same donor, typical of at least 3 separate donors (b).

**Figure 6:**
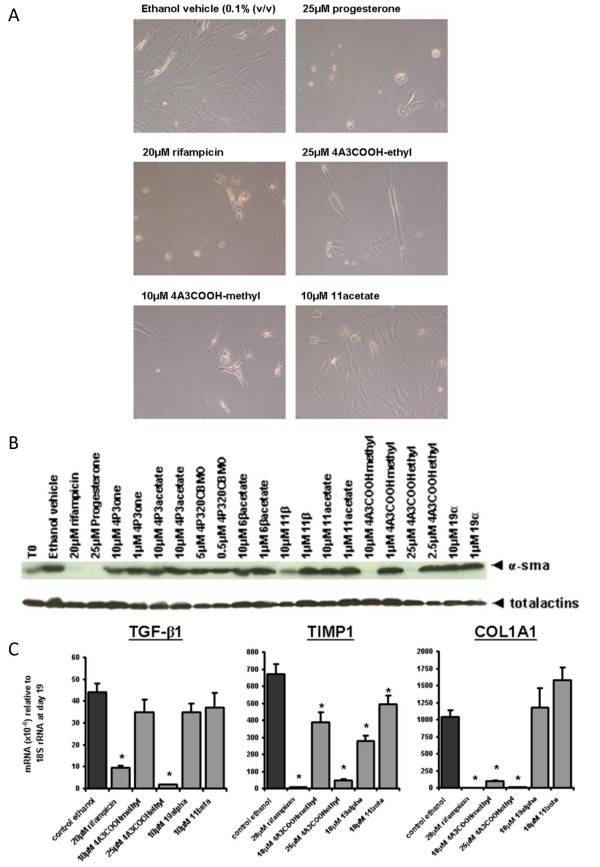
**Screening for inhibitors of trans-differentiation in rat and human HSCs – Part 2**. Photomicrographs of human HSCs at day 12 of culture with the indicated treatment (a); levels of expression of α-smooth muscle actin (α-sma) (b) and levels of expression of fibrogenic marker mRNA transcripts in human HSCs after treatment selected compounds for 19 days. Data are the mean and standard deviation relative transcript level from 3 separate treatments on cells from the same donor, typical of at least 3 separate donors (c).

HSCs are a major source of liver myofibroblasts in chronic liver injury and undergo a phenotypically-similar process of trans-differentiation *in vitro *when cultured on plastic in serum-containing medium [1]. Early screening for potential anti-fibrogenic compounds is commonly performed using this *in vitro *system [1]. PCN inhibited trans-differentiation as previously reported [6], whereas the other potent PXR activators were less effective (Fig. 5a). Interestingly, non-physiologically high levels of progesterone markedly inhibited rat HSC trans-differentiation, whereas substitution at the 11 position of progesterone had minimal effects on rPGRMC1 binding (Additional file 1) but abrogated the inhibitory effects of progesterone on trans-differentiation (Fig. 5a). A number of other compounds were also able to inhibit the trans-differentiation of rat HSCs (Fig 5a). However, when examined using human HSCs, only the PXR activator rifampicin (as previously reported [8]), progesterone, 11β hydroxyprogesterone, and 4 androstene-3-one 17β-carboxylic acid methyl ester (4A3COOHmethyl) showed significant inhibitory activity on trans-differentiation (Fig. 5b and 6).

Examination of myofibroblast expression of the major pro-fibrogenic cytokine TGFβ; the fibrogenic TIMP1 and collagen 1A1 mRNAs in human myofibroblasts treated with selected compounds showed that the PXR activator rifampicin (as previously reported [8]) and the PGRMC1 ligand 4A3COOHmethyl inhibited the expression of all mRNAs, whereas other PGRMC1 ligands were less effective (Fig. 6c).

### Effect of administration of 4A3COOHmethyl in an animal model of liver fibrosis

We selected 4A3COOHmethyl for use in an *in vivo *study for anti-fibrogenic activity, since this compound showed no activity as a PXR activator in either rat or human; competed with dexamethasone for binding to LAGS and was effective as a potential anti-fibrogenic in rat and human screens, *in vitro*. Since there was no information in the literature regarding any potential adverse effects of 4A3COOHmethyl administration, a pilot toxicity study was initially undertaken, in which adult male rats were administered 4A3COOHmethyl for 3 days at up to 100 mg/kg body weight by *i.p*. injection. Twenty four hours after the final treatment, liver serum enzyme levels and liver pathology were examined and no adverse effects were observed (data not shown).

To examine the effects of 4A3COOHmethyl on fibrosis, adult male rats were treated with 20 mg/kg body weight by *i.p*. injection every week during an 8 week twice weekly treatment with CCl_4_, to generate liver fibrosis. A reduced dose of 20 mg/kg body weight was chosen because the compound was to be administered to rats with compromised liver function. To avoid potential interactions with CCl_4_, toxicity (*i.e*., reductions in CCl_4 _hepatotoxicity that could be misinterpreted as anti-fibrogenic effects), 4A3COOHmethyl was not administered within a 48 hour period of CCl_4 _administration. Previous work has established that a similar dose of PCN – using the same dosing regimen – is sufficient to modulate fibrosis in animal models of fibrosis [6].

**Figure 7:**
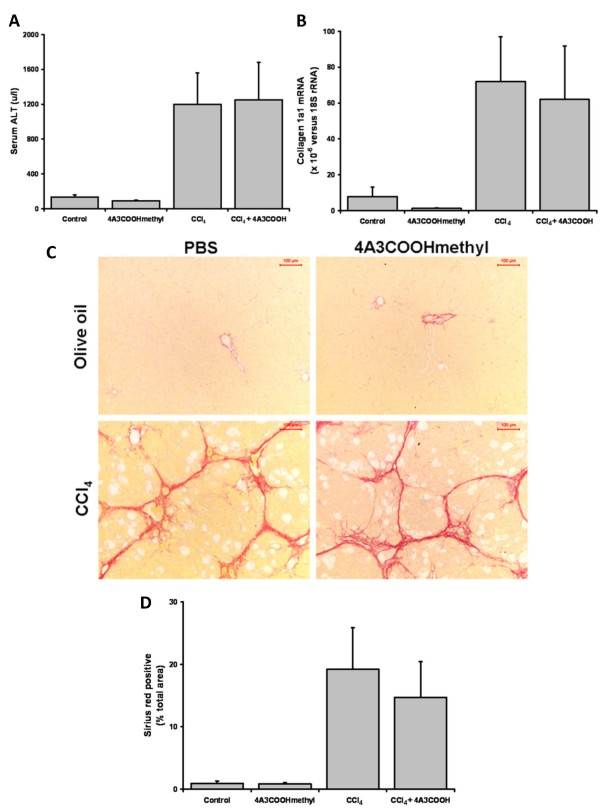
**4A3COOHmethyl administration and liver fibrosis in a rat CCl_4 _model of liver fibrosis**. Four animals/group (control or 4A3COOHmethyl) or six animals/group (CCl_4 _or CCl_4 _+ 4A3COOHmethyl) were treated as outlined in the Methods section. Mean and standard deviation serum ALT (a); Mean and standard deviation collagen 1A1 mRNA levels (b); typical views of liver sections stained for sirius red, with a 100 μm scale bar (b); quantitative image analysis for fibrosis – data are the mean and standard deviation percentage sirius red staining from at least 4 separate animals in each treatment with at least 10 randomly selected fields examined for each animal (c).

**Figure 8:**
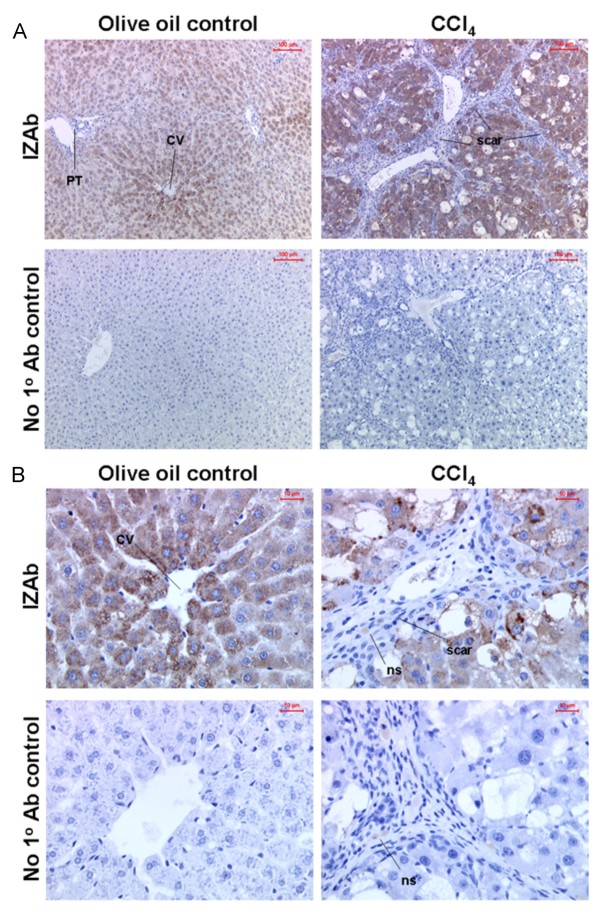
**Rat liver myofibroblast do not express rPGRMC1 *in vivo *– Part A**. Low power views (a) and high power views (b) of liver section immunohistochemically stained for rPGRMC1 using IZAb upper panels or identical staining without addition of IZAb (no 1° Ab control) from olive oil control or CCl_4 _treated animals (note CCl_4 _+ 4A3COOHmethyl treated animals gave similar results). PT, portal tract; CV, central vein; scar, primary location of scar matrix and liver myofibroblasts; ns non-specifically bound secondary antibody.

**Figure 9:**
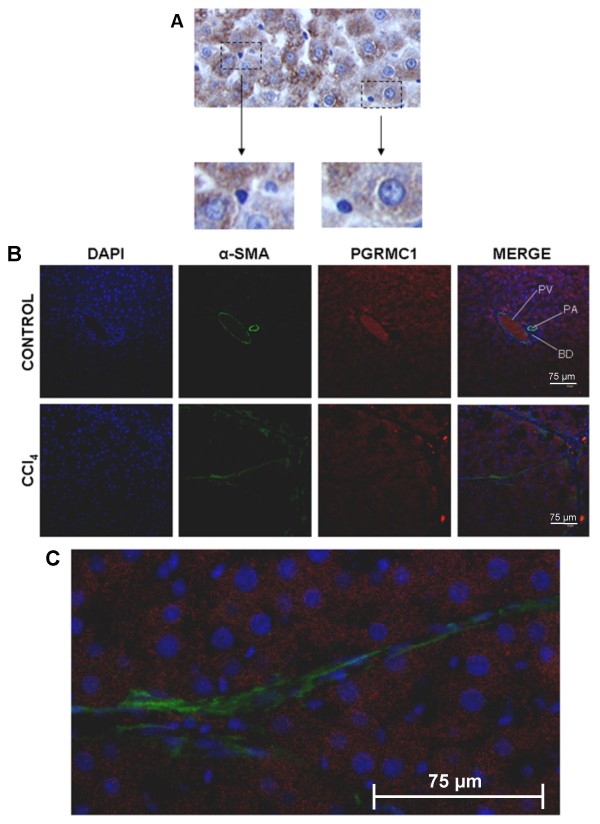
**Rat liver myofibroblast do not express rPGRMC1 *in vivo *– Part B**. High powered views show positive staining of non-parenchymal cells in control liver sections (a); co-staining sections from indicated treatment groups – DNA with DAPI (blue), α-sma (green) and PGRMC1 with IZAb (red) with merged panel (b); high powered view of merged liver section from CCl_4_-treated rat liver (c). PV, periportal venule; PA, periportal arteriole; BD, bile duct.

Figure 7a indicates that 4A3COOHmethyl administration did not affect serum levels of ALT after 8 weeks confirming that 4A3COOHmethyl did not inhibit the toxicity of CCl4. However, immunohistochemical α-smooth muscle actin staining for liver myofibroblasts (data not shown), determination of collagen 1a1 mRNA levels (Fig. 7b) and a staining for scarring extracellular matrix protein (Fig. 7c and 7d) indicate that 4A3COOHmethyl also did not significantly affect fibrosis severity. Liver sections were therefore immunostained for the presence of rPGRMC1 in vivo using the IZAb. Figures 8a and 8b (high power) indicates that rPGRMC1 expression showed an enhanced centrilobular pattern of expression in hepatocytes with clear evidence of expression in non-parenchymal cells such as quiescent HSCs in control liver sections (Fig. 9a), but not in bile duct epithelium (Fig. 8a). However, in CCl4-treated rat liver sections, there was little evidence for expression of rPGRMC1 in cells within the scar region other than likely non-specific binding of secondary antibody to occasional inflammatory cells, whereas hepatocytes showed enhanced expression (Fig. 8a and 8b). To firmly establish that rat liver myofibroblasts in vivo do not express rPGRMC1, fibrotic liver sections were co-stained for the expression of α-smooth muscle actin and rPGRMC1. Figure 9b and 9c shows that there was no co-staining of α-smooth muscle actin in liver myofibroblasts with rPGRMC1, which was restricted to hepatocytes in fibrotic liver sections. Identical staining was obtained in sections from animals treated with CCl4 or CCl4 and 4A3COOHmethyl (data not included).

## Discussion

Steroid hormone interaction with nuclear receptor proteins has been characterized over several decades. Steroids pass through plasma and/or nuclear membranes and interact with intracellular receptor proteins from the steroid/nuclear receptor gene super-family (such as the PXR), representing the canonical (genomic) mode of action for steroid hormone signalling [30]. Those proteins are ligand-modulated transcription factors and interact with specific DNA "response element" sequences as part of a co-ordinated regulation of gene expression [30]. In this way, steroid hormones modulate the expression of genes containing the required response element within their promoters in those cells which express the binding nuclear receptor. Nuclear receptors are associated with soluble fractions of cell. Nevertheless, steroids also interact in a specific and saturable manner with proteins in cell membranes [31]. The identity of these proteins (including PGRMC1) has only recently been determined and their function(s) remain to be fully established [32]. Over the years, it has been proposed that those proteins are associated with the non-genomic effects of steroid hormone action [32]. Steroid hormone-mediated changes in gene expression typically take in the order of hours for a change to be measurable. However, steroids also stimulate rapid (within seconds) changes in cells, such as alterations in calcium homeostasis [32]. These effects occur too fast to be dependent on changes in gene expression and have been suggested to be dependent on membrane-associated receptors and/or proteins such as PGRMC1 [32].

The data in this paper suggest that PGRMC1 is a steroid binding protein in agreement with Peluso *et al *[14]. However, neither our data nor the latter authors' data demonstrate binding with purified PGRMC1, leaving open the possibility that PGRMC1 is required for a functional steroid binding complex but may not be the direct binding protein within the complex. Procaryotic expression of PGRMC1 has failed to generate a binding species although this may be explained by the requirement for a eucaryotic-specific folding and/or post-translation modification. We have previously shown that phosphorylation of a truncated human PGRMC1 leads to steroid binding activity [9], and this may be crucial for effective and efficient binding of steroids by PGRMC1 or an associated protein. However, we have been unable to efficiently generate a binding protein in COS-7 cells most likely because the phosphorylation event is not efficiently mimicked or is rapidly reversed by de-phosphorylation. Accordingly, we had to rely on liver microsomal LAGS activity for our screening assays.

The function of PGRMC1 remains elusive and therefore the role that this protein plays in liver myofibroblasts can only be postulated. PGRMC1 shares close homology with the yeast protein Dap1p which is required for cell cycle progression following DNA damage [33]. PGRMC1 also protects cancer cells from oxidative damage [34]. More recently, PGRMC1 has been shown to bind haem and to modulate the activity of some cytochrome P450s [15]. The data in this paper demonstrate that a steroidal ligand for the LAGS/PGRMC1 potently inhibits the trans-differentiation of HSCs to fibrogenic myofibroblasts *in vitro*. The pivotal signal that directs HSC trans-differentiation has not been unequivocally identified; nonetheless, oxidative stress is known to be a promoter and possibly an essential component [1]. Since PGRMC1 protects cancer cells from oxidative stress [34], it is tempting to speculate that 4A3COOHmethyl modulates the ability of PGRMC1 to function in this capacity. Cytochrome P450s and the cytochrome P450 electron transport chain are a prominent source of reactive oxygen species, since their catalytic function involves the NAD(P)H-dependent splitting of molecular oxygen with concomitant mono-oxygenation of substrate and reduction to water. Cytochrome P450s such as the CYP51A1 – which are expressed in liver myofibroblasts *in vitro *[16] – may therefore be a source of reactive oxygen species that trigger HSC differentiation. Modulating the generation of reactive oxygen species through PGRMC1-mediated effects on cytochrome P450s may then be the mechanism of action by which 4A3COOHmethyl and other PGRMC1/LAGS ligands operate. Alternatively, 4A3COOHmethyl may modulate the levels of sterols generated by CYP51A1 or other cytochrome P450s that regulate trans-differentiation.

The 4A3COOHmethyl administration had no detectable effect on fibrosis, *in vivo*, using the rat CCl_4 _model of liver fibrosis. There are many potential reasons why this compound failed to demonstrate an anti-fibrogenic effect *in vivo*. The compound may not have achieved the required therapeutic concentrations *in vivo *because of absorption, distribution, metabolism or excretion effects that are not mimicked in the *in vitro *model employed. However, it is essential in these studies, to avoid any interaction with the injuring agent to avoid inadvertently identifying an anti-fibrogenic when in fact the agent is simply reducing injury. This consideration restricts potential anti-fibrogenic dosing periods to an extent although studies using the same protocol have been adequate to demonstrate anti-fibrogenic efficacy with other compounds [6,35]. It is notable that liver myofibroblasts are located adjacent to hepatocytes, *in vivo*, the most metabolically active cells toward drugs/xenobiotics in the body [1]. Hepatocytes actively sequester and metabolize a vast array of drugs/xenobiotics and therefore may reduce sufficiently the levels of anti-fibrogenic required to modulate myofibroblast activity. Thus, there may be a need in many instances for drugs to be directly targeted to myofibroblasts for the drug to be an effective anti-fibrogenic. In this respect, a number of targeting therapies are being developed including modified albumins that are sequestered by myofibroblasts [36-39] to antibodies that interact with a surface antigen on myofibroblasts [40-42]. However, evidence presented in this paper strongly suggest that PGRMC1 is not expressed in rat liver myofibroblasts, *in vivo*. Myofibroblasts may be derived from a number of sources *in vivo *including HSCs, the bone marrow and from epithelial-mesenchymal transition [1,43], whereas myofibroblasts generated *in vitro *are primarily derived from vitamin A-loaded quiescent HSC. So, few liver myofibroblasts may be derived from HSCs in the CCl_4 _model. A more likely scenario, however, is that HSC-derived myofibroblasts are not identical to liver myofibroblasts, *in vivo*. Previous work by others has shown that culture activation of HSCs into myofibroblasts only partially reproduced the gene expression changes observed during BDL- and CCl_4_-induced activation [44].

## Conclusion

Although 4A3COOHmethyl potently inhibited HSC trans-differentiation to pro-fibrogenic myofibroblasts *in vitro *without activating the PXR, it failed to inhibit liver fibrosis in an *in vivo *rat model. The cause of the disparity between *in vitro *and *in vivo *responses to 4A3COOHmethyl was most likely associated with a lack of expression of the PGRMC1 target in liver myofibroblasts *in vivo *in contrast to *in vitro *activated myofibroblasts. This underscores the importance of animal models for testing potential anti-fibrogenics and suggests that confirming the presence of drug targets *in vivo *(including human diseased liver tissue) may assist in the development of effective anti-fibrotic drugs for clinical use. These data also demonstrate that the anti-fibrogenic effects of PCN *in vivo *are likely mediated entirely *via *the PXR.

## Methods

### Reagents

All compounds in Additional files 1 and 2 were purchased from Steraloids (Rhode Island, USA) except dexamethasone, betamethasone, progesterone, androstenedione and testosterone which were purchased from the Sigma Chemical Company (Poole, UK). All other reagents were from local commercial sources and were of the highest purity available.

### Isolation and culture of HSCs

HSCs were isolated from rats (250–300 g body weight, Harlan, UK) by sequential pronase/collagenase perfusion of the liver followed by density gradient centrifugation and elutriation as previously outlined [45]. Human HSCs were isolated by an essentially similar procedure [46] using discarded tissue from patients undergoing a hepatectomy with patient consent and ethical approval by the Grampian Regional Ethical Committee. HSCs were seeded onto plastic culture dishes and cultured in Dulbecco's modified Eagle Medium (DMEM) containing 4.5 g/l of glucose and supplemented with 5% or 16% (v/v) fetal calf serum (for rat or human respectively), 80 μ/ml penicillin, 80 μg/ml streptomycin and 32 μg/ml gentamycin. Additional treatments were made by addition of compounds in an ethanol vehicle (stock solutions at 1000× final concentration). Ethanol at 0.1% (v/v) acted as control. Frequency of treatment (3 treatments per week/2 medium changes per week), as previously described [8].

### Isolation and culture of hepatocytes

Rat hepatocytes were prepared by collagenase perfusion, essentially as previously described [46,47], and cultured in William's Medium E supplemented with 1 μg/ml bovine insulin, 10% foetal calf serum (FCS), 80 μ/ml penicillin and 80 μg/ml streptomycin on collagen type-I coated 6 well plates (BD Biosciences). After 2 hours, the medium was renewed without FCS and insulin supplementation and thereafter changed daily with renewed media additions where indicated. Human hepatocytes were obtained from the UK Human Tissue Bank under MREC approval and were cultured in William's Medium E supplemented with 80 u/ml penicillin and 80 μg/ml streptomycin on collagen type-I coated plates. Addition treatments were made daily from ethanol stocks, essentially as outlined for HSCs above.

### Confocal microscopy

Cultured cells were fixed, as previously outlined [42], and incubated with primary antibodies – IZAb [23] and anti-CYP2E1 – followed by rhodamine red-conjugated anti-mouse IgG and FITC-conjugated anti-rabbit IgG (purchased from the Jackson Labs) to detect bound primaries, respectively. Cells were then examined using an Olympus BX50W1 microscope fitted with a Biorad μRadiance confocal scanning system and green (emission 515–530 nm) and red (emission > 570 nm) images captured. Staining without addition of primary antibodies was used to determine background fluorescence.

### RT-PCR and cloning rPGRC1

RNA was isolated using TRIzol (Invitrogen, Paisley, UK) according to manufacturer instructions and reversed transcribed using downstream primers and MMLV reverse transcriptase (Promega, Southampton, UK). The rPGRMC1 was amplified (35 cycles @ 52°C annealing temperature) using ratp28US (5'-TTTGCTCCAGAGATCATGGCT) and ratp28DS (5'-ACTACTCTTCAGTCACTCTTCCG) primers to amplify a 611 bp product. The human PGRMC1 was amplified (35 cycles @ 44°C annealing temperature) using hLAGSUS (5'-ATCATGGCTGCCGAGGATGTG) and hHPR6.6DS (5'-CACTGAATGCTTTAATCATTTTTCCGGGC) primers to amplify a 602 bp product. The rPGRMC1 PCR product includes the full amino acid sequence of the protein and was initially inserted into the pUniblunt TOPO vector (Invitrogen, Groningen, The Netherlands) and sequenced to check integrity. The sequence was identical to that previously published [21]. The rPGCMR1 insert was then sub-cloned into the pSG5 eucaryotic expression vector (Stratagene, La Jolla, USA) at the *Eco*RI site. Correctly oriented inserts were screened initially using *Bam*HI and *Nsi*I restriction and a selected clone (pSG5-rPGRMC1) confirmed by sequencing.

### Transfections and COS-7 cell binding assays

COS-7 cells were transfected at 30–50% confluency using Effectene transfection reagent (Qiagen, Southampton, UK) essentially according to the manufacturer's instructions with either pSG5 empty vector, pSG5-rPGRMC1 or the β-galactosidase-encoding pcDNA3.1e/lacZ vector (Invitrogen, Paisley, UK). Thirty hours after transfection, β-galactosidase activity was determined in fixed cells,*in situ*. Briefly, the culture medium was aspirated from the dish and the cells washed twice with PBS buffer (10 mM phosphate buffer, 2.7 mM KCl and 137 mM NaCl pH 7.4). The cells were then fixed in 2% (w/v) formaldehyde/0.2% (w/v) glutaraldehyde for 15 minutes followed by 3 washes in PBS buffer. The cells were then incubated with 1 mg/ml X-gal (5-bromo-4-chloro-3-indoyl β-D-galactoside) in PBS containing 4 mM K_3_Fe(CN)_6_, 4 mM K_4_Fe(CN)_6 _and 2 mM MgCl_2_. After incubation for 2 hours at 37°C, β-galactosidase positive (blue) cells were identified by microscopic examination of 10 randomly selected fields. For the determination of steroids binding activity, the medium was discarded and the cells were washed twice with ice-cooled HBSS (0.14 M NaCl, 5.4 mM KCl, 0.34 mM Na_2_HPO_4_, 0.44 mM KH_2_PO_4_, 5.6 mM glucose, 1 mM CaCl_2_, 6 mM HEPES, 4 mM NaHCO_3 _pH 7.4). Cells were then harvested using a cell scraper and pelleted by centrifugation. Steroid binding activity was determined in homogenised COS-7 cell extracts prepared by re-suspending cell pellets in 10 mM Tris, 250 mM sucrose pH 7.4 buffer and disruption using a Turrax homogenisor. The homogenate was then centrifuged at 13,000 g for 5 minutes at 4°C. The supernatant was retained and assayed for protein concentration using the method of Lowry and binding activity using 100 nM [^3^H]dexamethasone with or without excess unlabelled dexamethasone. After overnight incubation on ice, free ligand was removed by charcoal dextran adsorption and bound ligand determined in supernatants by liquid scintillation essentially, as previously described [9-11].

### Westerns

Western Blotting was performed after SDS-PAGE under reducing conditions using a MiniP2 Biorad electrophoresis apparatus. Protein was transferred onto nitrocellulose and blocked overnight with 3% (w/v) milk protein/0.3% (w/v) Tween 20. Antibody raised against the C-termini of CYP3A1/3A23 (IITGS) was used, as described previously [11]. The anti-α-smooth muscle actin and anti-β-actin (cross reacts with all actin isoforms) antibodies were purchased from the Sigma Chemical Co (Poole, UK) and Chemicon (Chandlers Ford, UK), respectively. The anti-CYP2E1 and anti-LAGS (IZ-Ab) antibodies were obtained from Prof. M. Ingelman-Sundberg, Karolinska Institutet, Stockholm, Sweden, and Prof. Gavin Vinson, Queen Mary College, London, UK. After incubation with primary antibodies, blots were incubated with the appropriate horseradish peroxidase conjugated anti-IgG antibody. Detection was accomplished using chemiluminescence with the ECL kit (Amersham).

### Microsomal receptor-ligand binding assay

Rat liver microsomes were prepared and incubated with [^3^H] dexamethasone to determine LAGS activity, as previously outlined [9-11]. In brief, rats were anaesthetized with pentobarbital and a 16G cannula inserted into the hepatic portal vein and secured. The blood was cleared from the liver by pumping ice-cooled perfusion buffer (0.14 M NaCl, 5.4 mM KCl, 0.34 mM Na_2_HPO_4_, O.44 mM KH_2_PO_4_, 15.7 mM NaHCO_3 _and 5.6 mM glucose, pH 7.4) through the liver at 50 mls per minute. The liver was then excised and chopped roughly with ice-cooled TS buffer (10 mM Tris/HCl pH 7.4 containing 250 mM sucrose) and disrupted using a Potter-Elvehjem homogenisor. The resultant homogenate was then centrifuged at 12,000 g for 20 minutes at 4°C and the supernatant retained and centrifuged at 100,000 g for 60 minutes at 4°C. The resultant "microsomal" pellet was re-suspended in fresh TS buffer and re-centrifuged at 100,000 g to obtain a washed microsomal pellet. The pellet was finally re-suspended in TS buffer supplemented with 5 mM dithiothreitol and used immediately (on ice at all times) by addition of [^3^H]dexamethasone with or without excess unlabelled dexamethasone. After overnight incubation on ice, free ligand was removed by charcoal dextran adsorption and bound radioligand determined in supernatants by liquid scintillation, essentially as previously described [9-11].

### Quantitative RT-PCR

Quantitative transcript expression was examined after reverse transcription (using a Taqman reverse transcription kit (Applied Biosystems)) using a PE Applied Biosystems ABI7700 and PE Applied Biosystems Gene Expression Assay™, incorporating sequence-specific forward, reverse and fluorescently labelled probes (see Table 2).

**Table 2:** qRT-PCR kits used in these studies.

Transcript mRNA	Applied Biosystems Primer Kit
Human	
18S rRNA	Hs99999901_sl
TGF-β1	Hs00171257_ml
TIMP1	Hs00171558_ml
COL1A1	Hs00164004_ml
	
Rat	
GAPDH	4352338E
COL1A1	Rn00801649_g1

### In vivo animal study and tissue analysis

Rats were administered CCl_4 _mixed 1:1 (v/v) with olive oil – 2 ml/kg body weight by *i.p*. injection – twice weekly for 8 weeks to generate liver fibrosis [46]. Control animals were administered olive oil alone (1 ml/kg body weight). Once a week between CCl_4 _treatment, rats received 4A3COOHmethyl (20 mg/kg body weight). After 8 weeks, animals were killed by cervical dislocation and samples of various tissues removed and fixed in 10% formalin in PBS. Blood was allowed to clot prior to centrifugation to obtain serum for analyses. Serum ALT levels, α-smooth muscle actin immunostaining and sirius red staining were performed as previously outlined [6,46].

## Competing interests

The authors declare that they have no competing interests.

## Authors' contributions

CJM performed most of the experiments, biochemical analyses and prepared the manuscript. KW performed the majority of the immunohistochemical staining, ED and VL cloned all constructs, MK prepared human tissue for experimentation, LJL performed some of the Western blotting and RT-PCR. MCW designed and supervised the studies. All authors read and approved the final manuscript.

## Supplementary Material

Additional file 1 **Supplemental table S1**. Competition of substituted progestins for binding to rat liver microsomes Click here for file

Additional file 2 **Supplemental table 2**. Competition of dexamethasone derivatives for binding to rat liver microsomes. Click here for file
