# Supplementary material for: Low affinity glucocorticoid binding site ligands as potential anti-fibrogenics
Source: Comp Hepatol. 2009 May 11;8:1. doi: 10.1186/1476-5926-8-1 (PMC2688476; doi:10.1186/1476-5926-8-1)
Supplement: Additional file 1 — Supplemental table S1. Competition of substituted progestins for binding to rat liver microsomes [file 1476-5926-8-1-S1.doc]

**Additional file 1. Competition of substituted progestins for binding to rat liver microsomes.**

| **Competitor** | **Abbrev.** | **Basic Structure** | **Substitution (R)** | **IC_50%_** |
| --- | --- | --- | --- | --- |
| Substitution at Posn 3 |  | 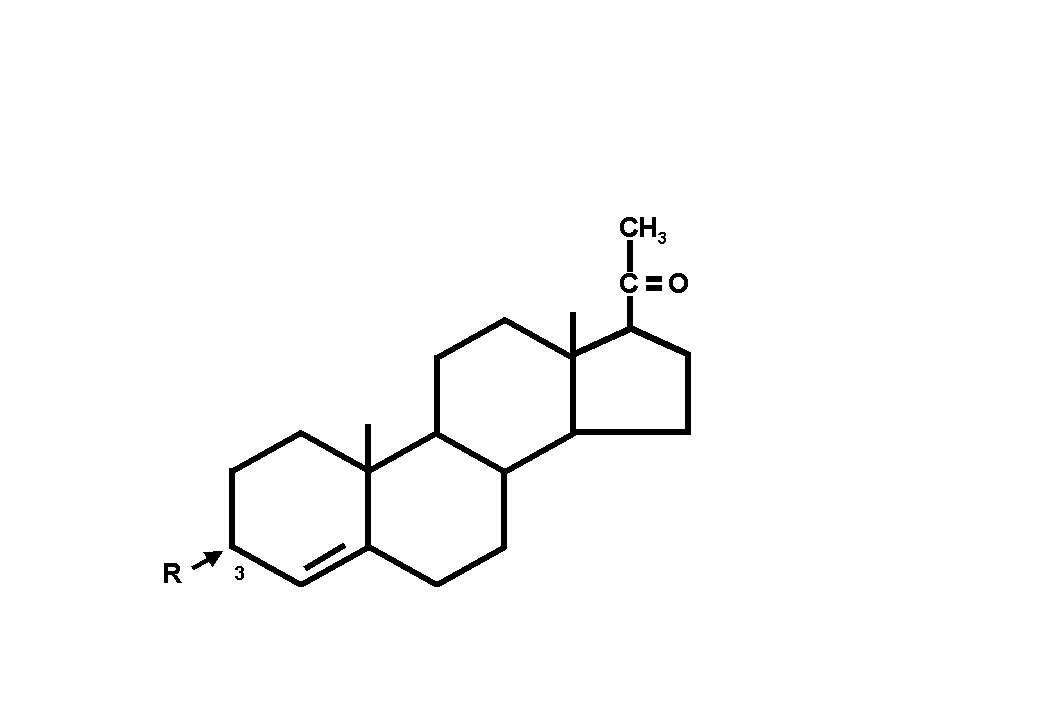 |  |  |
| progesterone | prog |  | O= | 50 nM |
| 4 pregnene-3β-ol-20-one | 4P3one |  | HO- | 500 nM |
| 4 pregnene-3β-ol-20-one acetate | 4P3acetate |  | CH_3_COO- | 500 nM |
| 4 pregnene-3β-ol-20-one carboxymethyloxime | 4P320-CBMO |  | HOOC-CH_2_-O-N= | >100 μM |
| Substitution at Posn 6 |  | 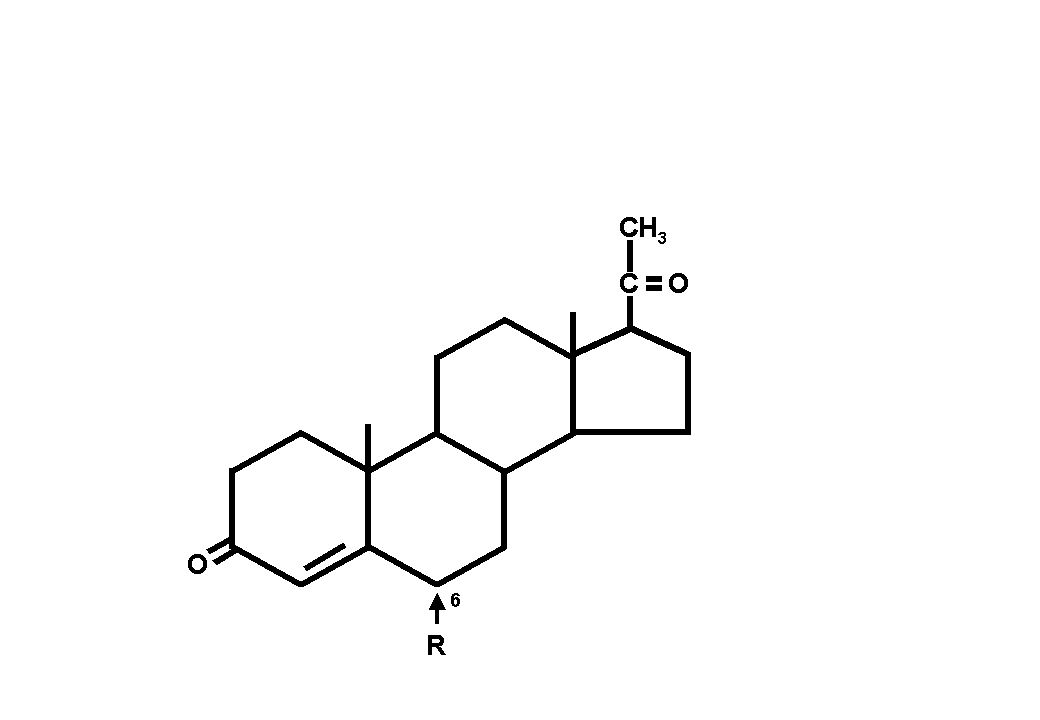 |  |  |
| progesterone | prog |  | H- | 50 nM |
| 6α-hydroxyprogesterone | 6α |  | HO- | 5 μM |
| 6β-hydroxyprogesterone | 6β |  | HO- | 10 μM |
| 6β-progesterone acetate | 6β-acetate |  | CH_3_-COO- | 500 nM |
| Substitution at posn 11 |  |  |  |  |
| progesterone | prog | 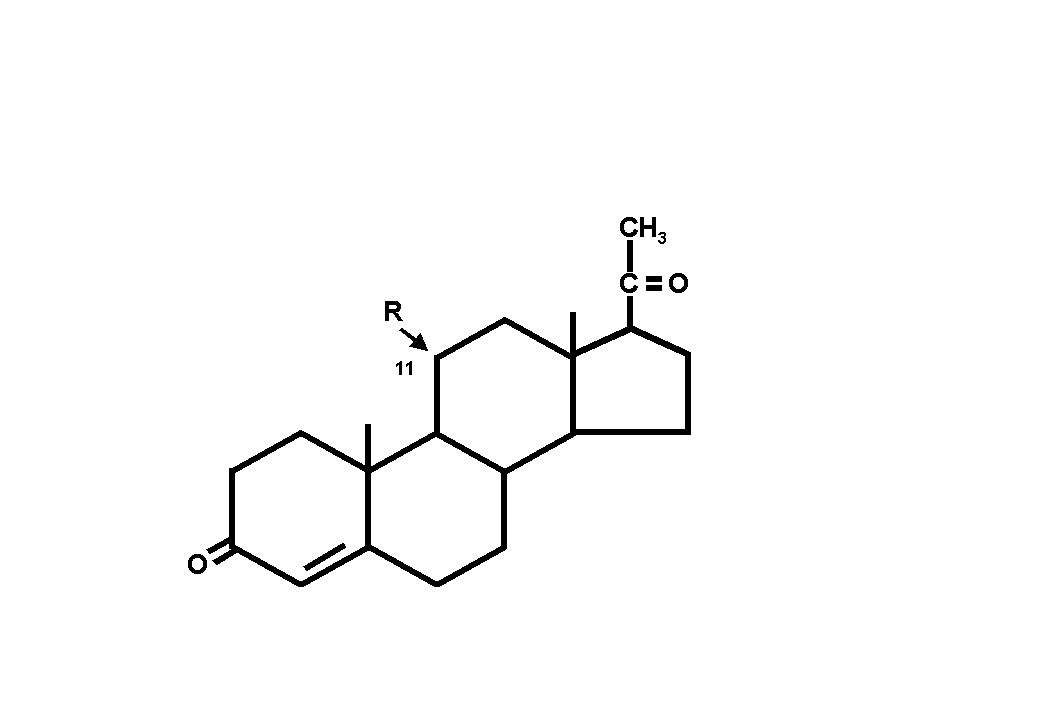 | H- | 50 nM |
| 11α-hydroxyprogesterone | 11α |  | HO- | 100 nM |
| 11β-hydroxyprogesterone | 11β |  | HO- | 50 nM |
| 11α-progesterone acetate | 11acetate |  | CH_3_-COO- | 100 nM |
| 11α-progesterone tosylate | 11tosylate |  | CH_3_-Bz-SO_3_- | 10 μM |
| Substitution at posn 17 |  |  |  |  |
| progesterone | prog | 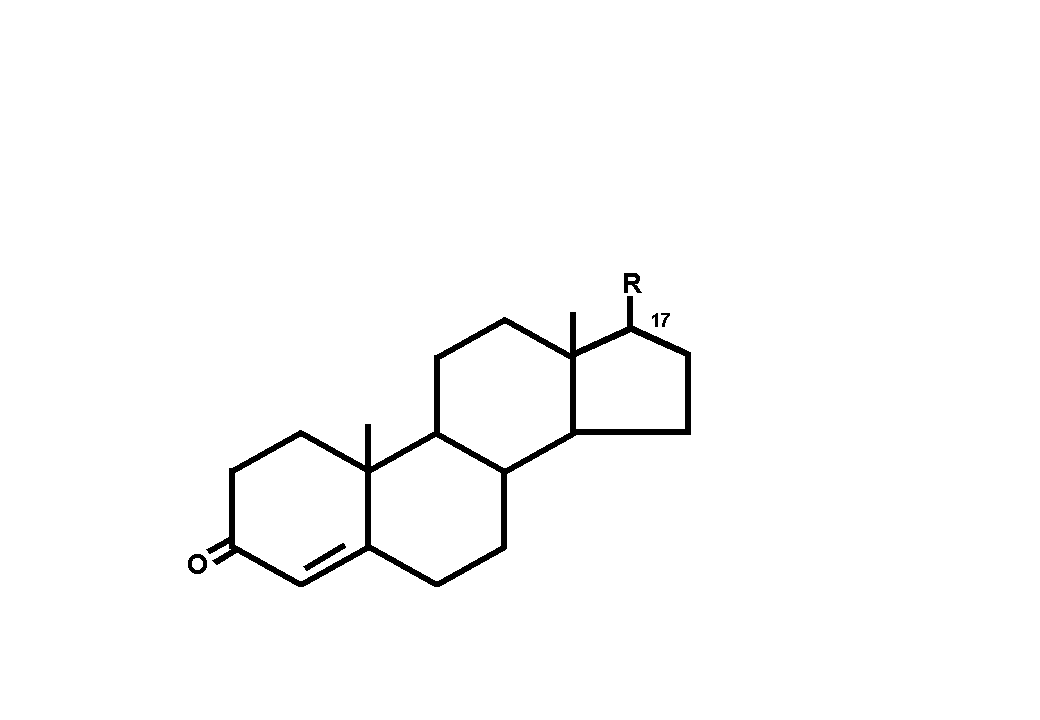 | CH_3_-CO- | 50 nM |
| androstenedione | ADD |  | O= | 5 μM |
| 4 androstene-3-one 17β-carboxylic acid methyl ester | 4A3COOHmethyl |  | CH_3_OOC- | 100 nM |
| 4 androstene-3-one 17β-carboxylic acid ethyl ester | 4A3COOHethyl |  | CH_3_CH_2_OOC- | 100 nM |
| testosterone proprionate | test-prop |  | CH_3_CH_2_COO- | 10 μM |
| testosterone | test |  | HO- | 10 μM |

Bz, Benxyl group.
