# Supplementary material for: Low affinity glucocorticoid binding site ligands as potential anti-fibrogenics
Source: Comp Hepatol. 2009 May 11;8:1. doi: 10.1186/1476-5926-8-1 (PMC2688476; doi:10.1186/1476-5926-8-1)
Supplement: Additional file 2 — Supplemental table 2. Competition of dexamethasone derivatives for binding to rat liver microsomes. [file 1476-5926-8-1-S2.doc]

**Additional file 2. Competition of dexamethasone derivatives for binding to rat liver microsomes.**

| **Competitor** | **Abbrev.** | **Basic Structure** | **Substitution (R)** | **IC_50%_** |
| --- | --- | --- | --- | --- |
| Dexamethasone / substitution at posn 17 |  |  |  |  |
| dexamethasone | dex | 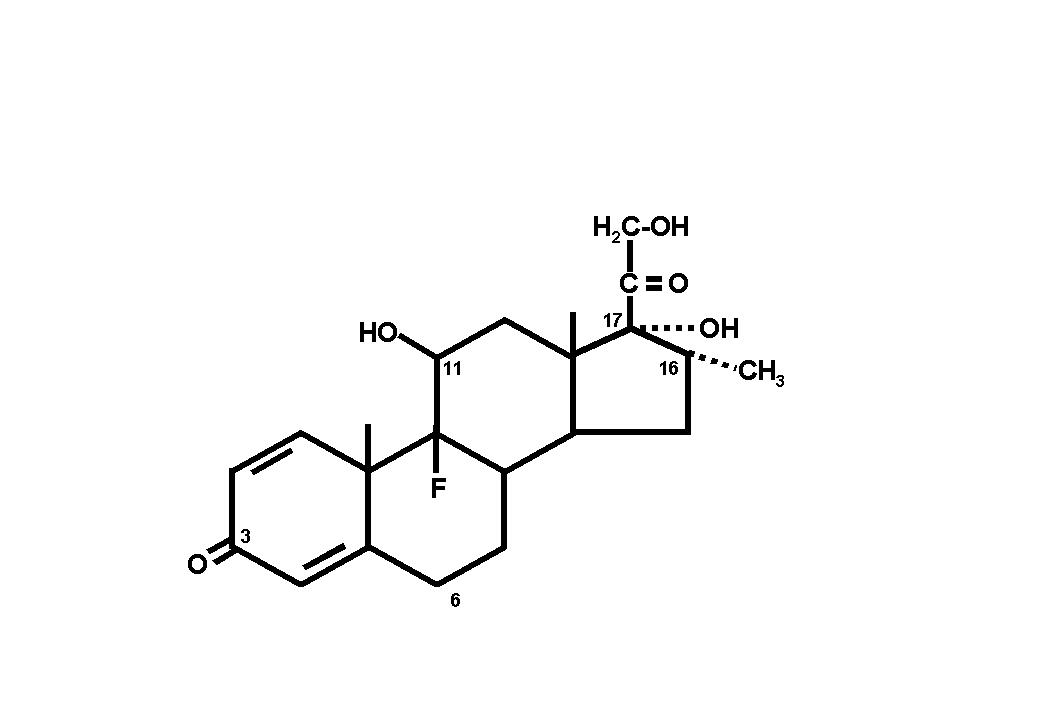 | HO-CH_2_-CO- | 50 nM |
| betamethasone | beta |  | HO-CH_2_-CO-  *CH_3_ at posn 16 is β configuration* | 5 μM |
| 17β-carboxylic acid derivative^*^ | 17βCOOH |  | HOOC- | 100 μM |
| biotinylated dexamethasone | dex-biotin |  | biotin-CO- | >100 μM |
| dexamethasone mesylate | dex-mes |  | CH_3_-SO_2_-O-CH_2_-CO- | 10 μM |

^*^9α-fluoro 16α-methyl-11β,17α-dihydroxy-1,4-pregn-3-one 17-carboxylic acid.
